# Hypertrophic cardiomyopathy in myosin-binding protein C (*MYBPC3*) Icelandic founder mutation carriers

**DOI:** 10.1136/openhrt-2019-001220

**Published:** 2020-04-05

**Authors:** Berglind Adalsteinsdottir, Michael Burke, Barry J Maron, Ragnar Danielsen, Begoña Lopez, Javier Diez, Petr Jarolim, Jonathan Seidman, Christine E. Seidman, Carolyn Y Ho, Gunnar Th Gunnarsson

**Affiliations:** 1 Department of Medicine, University of Iceland, Reykjavik, Iceland; 2 Division of Cardiology, Landspitali - The National University Hospital of Iceland, Reykjavik, Iceland; 3 Department of Genetics, Harvard Medical School, Boston, Massachusetts, USA; 4 Cardiology Division, Emory University School of Medicine, Atlanta, Georgia, USA; 5 Hypertrophic Cardiomyopathy Center, Division of Cardiology, Tufts Medical Center, Boston, Massachusetts, USA; 6 Program of Cardiovascular Diseases, Centre for Applied Medical Research, University of Navarra, Pamplona, Navarra, Spain; 7 Carlos III Health Institute, Madrid, Spain; 8 Department of Pathology, Brigham and Women's Hospital, Boston, Massachusetts, USA; 9 Cardiovascular Division, Brigham and Women's Hospital, Boston, Massachusetts, USA; 10 Howard Hughes Medical Institute, Boston, Massachusetts, USA; 11 Department of Medicine, Akureyri Hospital, Akureyri, Iceland

**Keywords:** cardiomyopathy hypertrophic, genetics, echocardiography

## Abstract

**Objective:**

The myosin-binding protein C (*MYBPC3*) c.927-2A>G founder mutation accounts for >90% of sarcomeric hypertrophic cardiomyopathy (HCM) in Iceland. This cross-sectional observational study explored the penetrance and phenotypic burden among carriers of this single, prevalent founder mutation.

**Methods:**

We studied 60 probands with HCM caused by *MYBPC3* c.927-2A>G and 225 first-degree relatives. All participants underwent comprehensive clinical evaluation and relatives were genotyped.

**Results:**

Genetic and clinical evaluation of relatives identified 49 genotype-positive (G+) relatives with left ventricular hypertrophy (G+/LVH+), 59 G+without LVH (G+/LVH−) and 117 genotype-negative relatives (unaffected). Compared with HCM probands, G+/LVH+ relatives were older at HCM diagnosis, had less LVH, a less prevalent diastolic dysfunction, fewer ECG abnormalities, lower serum N-terminal pro-B-type natriuretic peptide (NT-proBNP) and high-sensitivity cardiac troponin I levels, and fewer symptoms. The penetrance of HCM was influenced by age and sex; specifically, LVH was present in 39% of G+males but only 9% of G+females under age 40 years (p=0.015), versus 86% and 83%, respectively, after age 60 (p=0.89). G+/LVH− subjects had normal wall thicknesses, diastolic function and NT-proBNP levels, but subtle changes in LV geometry and more ECG abnormalities than their unaffected relatives.

**Conclusions:**

Phenotypic expression of the Icelandic *MYBPC3* founder mutation varies by age, sex and proband status. Men are more likely to have LVH at a younger age, and disease manifestations were more prominent in probands than in relatives identified via family screening. G+/LVH− individuals had subtle clinical differences from unaffected relatives well into adulthood, indicating subclinical phenotypic expression of the pathogenic mutation.

**Key questions**

**What is already known about this subject?**

- Hypertrophic cardiomyopathy (HCM) is the most common monogenic heart disease, with diverse genetic and phenotypic expression. More than 2000 causative mutations in at least eight genes have been described in association with HCM. Although mutations in myosin-binding protein C (*MYBPC3*) represent 30%–40% of all HCM mutations, most of the causal mutations are usually unique to one or few families.

**What does this study add?**

- A prevalent *MYBPC3* founder mutation is responsible for >90% of sarcomere HCM in Iceland, providing valuable opportunity to explore associations between genotype and clinical phenotype. To our knowledge, this is the largest and most comprehensive genotype–phenotype study that exists on a single HCM mutation. Phenotypic manifestations of the Icelandic *MYBPC3* founder mutation varied by age, sex and proband status, and were remarkably similar to other distinct founder populations with truncating *MYBPC3* variants, suggesting a common effect in the context of haploinsufficiency in the myosin-binding protein.

**How might this impact on clinical practice?**

- Our findings underscore the need for lifelong surveillance of male and female *MYBPC3* mutation carriers, and identifying factors that modify disease expression and drive phenotypic progression.

## Introduction

Hypertrophic cardiomyopathy (HCM) is a genetically heterogeneous disorder with highly variable clinical expression. Although most HCM mutations occur in two cardiac sarcomere protein genes, *MHY7* (encoding β-myosin heavy chain) and *MYBPC3* (encoding myosin-binding protein C), specific pathogenic variants are usually unique to one or a few families. The small numbers of individuals with identical HCM mutations, the great diversity in background genotypes and the substantial phenotypic heterogeneity has limited the ability to determine the impact of genotype on phenotype and clinical outcomes. Studying populations with founder mutations helps address some of these challenges, but to date, only a few analyses have systematically described HCM phenotypes caused by founder mutations in at-risk relatives.^1 2^

We previously reported an Icelandic *MYBPC3* founder mutation, c.927-2A>G, that was introduced ~500 years ago and has an estimated population prevalence of 0.36%. This single mutation is responsible for >90% of sarcomeric HCM in Iceland,^3^ a geographically isolated country with a relatively homogeneous population. Here, we explored the inheritance and clinical consequences of the Icelandic *MYBPC3* founder mutation in 60 HCM probands and 225 first-degree relatives. Because the prevalence of the Icelandic variant is higher than that seen in other single founder variants,^1 4–7^ it provides a valuable opportunity to explore associations between genotype and clinical phenotype at a larger scale.

## Methods

### Study design and population

This study used a cross-sectional observational cohort design. HCM probands had a previously established HCM diagnosis and were found to carry the *MYBPC3* c.927-2A>G founder mutation through a nationwide genetic study of HCM in Iceland.^3^ deCODE Genetics genealogical database was used to identify all first-degree relatives. None of the relatives were previously diagnosed with HCM. Relatives were grouped according to genotype and the presence or absence of left ventricular hypertrophy (LVH; defined as a LV wall thickness (LVWT) ≥12 mm in adults or a z-score of ≥2 in children) as follows: mutation carrier with LVH (G+/LVH+), mutation carrier without LVH (G+/LVH−) and healthy mutation-negative (unaffected).

Participants underwent study visit between November 2012 and June 2014, including assessment of medical history and heart failure symptoms (New York Heart Association functional class), blood pressure, ECG and standardised echocardiography. Blood was drawn for genetic analysis and serum biomarker analysis. Subjects with known hypertension, cardiovascular disease other than HCM or medical conditions associated with increased collagen turnover were excluded. All investigators were blinded to the subjects’ clinical and genetic status. This study complied with the Declaration of Helsinki and was approved by the National Bioethics Committee of Iceland and the Icelandic Data Protection Authority. Informed consent was obtained from all participants, and parental consent was obtained for participants under 18 years of age.

### Genetic analysis

Genomic DNA, extracted from whole-blood samples, was obtained from all relatives and genotyped to determine the presence or absence of the *MYBPC3* c.927-2A>G mutation, as previously described.^3^

### Echocardiography

Transthoracic echocardiograms were obtained using Vivid E9 ultrasound systems (GE Healthcare, Horten, Norway), including two-dimensional images, spectral, colour and tissue Doppler interrogation following a standardised protocol. The average of three cardiac cycles was used for measurements of cardiac dimensions, mitral inflow patterns and myocardial velocities according to published guidelines.^8 9^ Maximal LVWT was defined by the maximal LVWT in any of the LV segments measured in the long-axis or short-axis parasternal view. Relative wall thickness was defined as two times the posterior wall thickness divided by the LV end-diastolic diameter (LVEDD). Left atrial (LA) volumes were measured using the biplane disk summation at end-systole. To allow comparisons between subjects with different body sizes, chamber measurements were reported as indexed to body surface area (BSA). The peak instantaneous LV outflow tract gradient was measured at rest and during the Valsalva manoeuvre with continuous-wave Doppler. The pattern of LV septal hypertrophy was divided into the following morphological subtypes: reverse curvature, sigmoid, neutral and apical.^10^ Diastolic function was assessed and graded as normal or diastolic dysfunction grades 1–3 based on expert consensus recommendations.^9^ Images were analysed offline using Echopac BT 112 software (GE Healthcare, Horten, Norway). Echocardiograms were analysed by two observers with expertise in cardiac imaging (BA, GTG) without knowledge of genotypes.

### ECG

Standard 12-lead ECGs were obtained at rest in the supine position. All ECGs were analysed by a single investigator (BA) according to standard criteria as described elsewhere.^11^ The Sokolow–Lyon LVH voltage criteria were defined as SV_1_ + RV_5_ or RV_6_ ≥35 mm. The Cornell LVH voltage criteria were defined as a RaVL+ SV_3_>28 mm for men and >20 mm for women. Conduction disturbance included interventricular conduction delay (QRS duration ≥110 ms), left bundle branch block or right bundle branch block. QST was defined as the presence of pathological Q waves, T wave inversion or ST-segment depression.^11^

### Serum biomarkers

Plasma concentrations of N-terminal B-type natriuretic peptide (NT-proBNP), high-sensitivity troponin I (hsTnI) and carboxy-terminal propeptide of procollagen type I (PICP) were measured. Plasma concentrations of NT-proBNP and hsTnI were measured in the Biomarker Research and Clinical Trials Laboratory at Brigham and Women’s Hospital, Boston, MA. NT-proBNP was measured using the proBNPII immunoassay (Roche, Indianapolis, IN, USA) with a coefficient of variation (CV) of 3.8% at 127 pg/mL and 2.4% at 4180 pg/mL. hsTnI was measured using an ultrasensitive immunoassay utilising a single-molecule counting technology (Erenna hsTnI, Singulex, Palo Alto, CA, USA) with a CV of 12.5% at 6.8 ng/L and 13.7% at 39.2 ng/L. PICP was measured at the CIMA of University of Navarra, Pamplona, Spain, using the METRA Enzyme Immunoassay (Quidel Corporation) with an inter and intra-assay CV of 6.3% and 6.4%, respectively, and a lower limit of detection of 0.2 ug/L. All assays were performed using commercially available reagents by technicians blinded to clinical and genetic statuses of the subjects. Details have been described previously.^12 13^

### Statistical analysis

Descriptive data are presented as the means±SD or counts and proportions. Baseline characteristics were compared with Student’s *t* test for continuous variables, and the χ^2^ test or Fisher’s exact test for categorical variables, as appropriate. Generalised linear models were used to examine differences in echocardiographic measures, ECGs and biomarkers, adjusting for age, sex and within-family correlation, where correlation between relatives was assumed to be proportional to their kinship. Values are expressed as the adjusted means (SE). Biomarkers were log-transformed due to skewed distributions, and values below the detectable threshold were set to the lower detection limit (0.1 pg/mL; n=9 for hsTnI). Pearson correlation coefficients were calculated to assess correlations between continuous variables. P values of <0.05 were considered statistically significant.

## Results

### Study population

**Figure 1:**
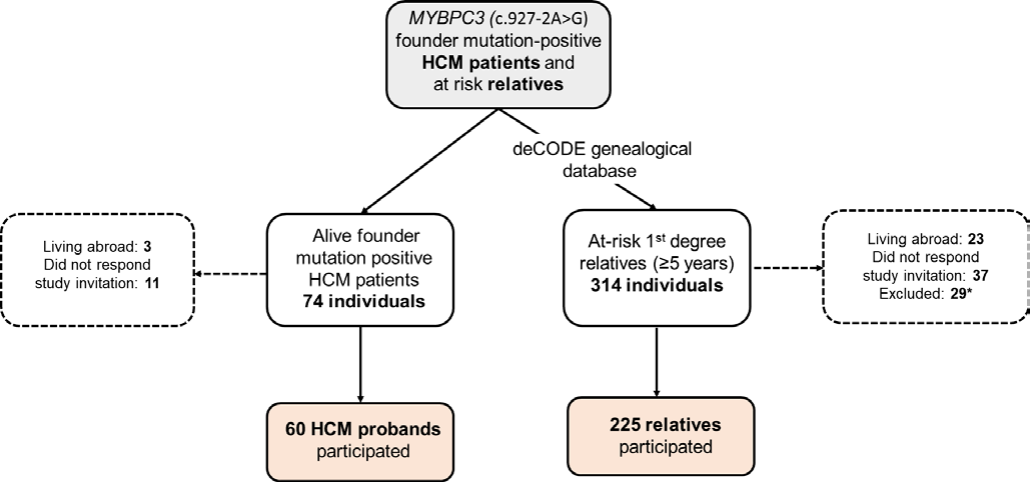
A flow diagram demonstrating the study cohort. *27 individuals were excluded due to hypertension and/or coronary artery disease, and 2 because of metastatic cancer.

We studied 285 individuals, including 60 HCM probands (mean age, 53±17 years) described previously^3^ and 225 first-degree relatives (figure 1). Of the relatives, 108 (48%) were founder mutation carriers (G+; mean age 41±20 years) and 117 (52%) were healthy mutation-negative individuals (unaffected; mean age, 40±18 years). Among the G+ relatives, 49 (45%) were G+/LVH+ (mean age, 50±19 years) and 59 were G+/LVH− (mean age, 34±17 years).

### Baseline clinical characteristics

Baseline demographics are summarised in table 1. HCM probands and G+/LVH+ relatives were predominantly men (67% and 55%, respectively). The probands were diagnosed with HCM at younger ages (41±14 years) than G+/LVH+ relatives (50±19 years; p value=0.004) and had more symptoms (48% HCM probands vs 4% G+/LVH+ relatives were New York Heart Association (NYHA) functional class ≥II; p<0.0001). Although the majority (96%) of G+/LVH+ relatives were asymptomatic, one (8.2%) had a history of atrial fibrillation, four (8.2%) had experienced unexplained syncope and one (2%) had been hospitalised for symptomatic heart failure.

**Table 1:** Baseline clinical characteristics by study group

	HCM probands	Relatives			P value HCM probands versus G+/LVH+ relatives	P value G+/LVH− versus unaffected
		G+/LVH+	G+/LVH−	Unaffected		
	(n=60)	(n=49)	(n=59)	(n=117)		
Age at enrolment, years	53±17	50±19	34±17	40±18	0.379	0.100
Age at HCM diagnosis, years	41±14	50±19			0.004	
Male	40 (67%)	27 (55%)	19 (32%)	55 (47%)	0.217	0.060
Reason for diagnosis						
Symptoms	24 (40%)					
Incidental finding*	22 (37%)					
Prior screening†	14 (23%)					
Body surface area (kg/m^2^)	2.01±0.22	1.99±0.23	1.76±0.30	1.92±0.36	0.982	0.169
Blood pressure, mm Hg						
Systolic	120±13	125±12	117±13	122±14	0.037	0.360
Diastolic	73±9	75±10	70±9	74±10	0.075	0.605
NYHA class					<0.0001	0.994
I	31 (52%)	47 (96%)	58 (98%)	115 (98%)		
II	26 (43%)	2 (4%)	1 (2%)	2 (2%)		
III	3 (5%)	0	0	0		
IV	0	0	0	0		
Medication use						
Amiodarone	5 (8%)	1 (2%)	0	0	0.152	
Anticoagulation	4 (7%)	1 (2%)	0	0	0.251	
ACE inhibitor/ARB	10 (17%)	0	0	0	0.021	
β-blocker	36 (60%)	11 (22%)	3 (5%)	0	<0.0001	0.036
Calcium channel blocker	6 (10%)	3 (6%)	0	0	0.464	
No cardiovascular medications	17 (28%)	37 (76%)	56 (95%)	117 (100%)	<0.0001	0.036
History of atrial fibrillation	11 (18%)	4 (8%)	0	0	0.126	
Heart failure hospitalisation	6 (10%)	1 (2%)	0	0	0.129	
History of stroke/TIA	0	0	0	1 (0.9%)		1.00
Unexplained syncope	11 (18%)	4 (8%)	0	0	0.135	
Implantable cardioverter defibrillator	11 (18%)	0	0	0	<0.0001	

Values are means±SD or numbers and proportions (%). P values for body surface area and blood pressure were adjusted for age and sex.

*12 had abnormal ECGs, 3 were examined because of heart murmurs and five underwent echocardiography due to other reasons.

†14 individuals were diagnosed through prior screening 14±9 years ago.

ACE, angiotensin-converting enzyme; ARB, angiotensin receptor blocker; G+, genotype positive; HCM, hypertrophic cardiomyopathy; LVH, left ventricular hypertrophy; NYHA, New York Heart Association; TIA, transient ischaemic attack.

G+/LVH− relatives were predominantly women (68%); 98% had no symptoms (NYHA class I) and none had a history of atrial fibrillation.

### Echocardiographic findings

**Figure 2:**
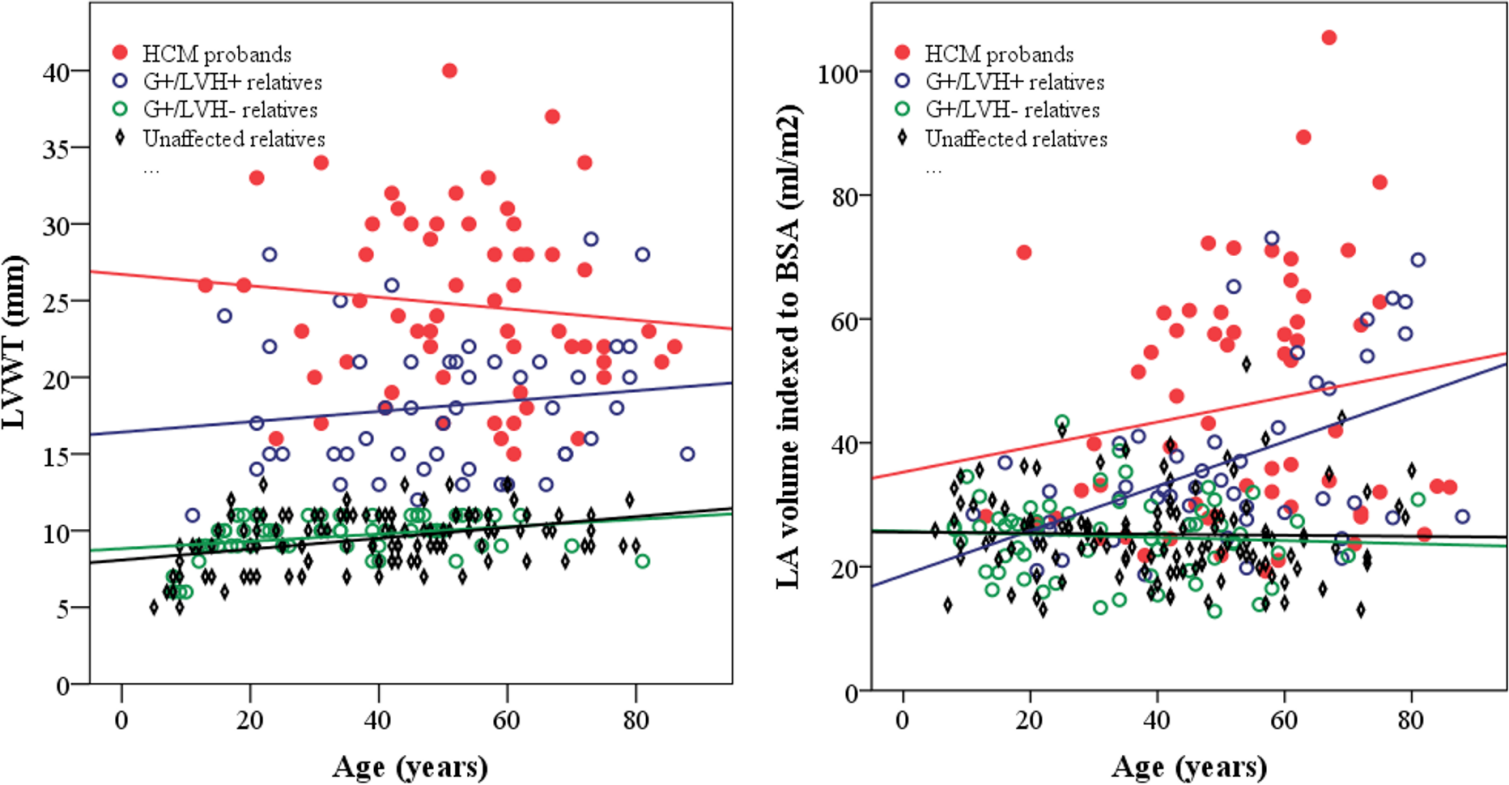
Scatter plots stratified by genotype–phenotype showing the relationship between (a) age and LVWT. There was no correlation between age and LVWT in HCM probands (red line, r=−0.11, p=0.42) and G+/LVH+ relatives (blue line, r=0.14, p=0.32). A modest linear increase in LVWT by age was noted in G+/LVH− relatives (green line, r=0.32, p=0.015) and unaffected relatives (black line, r=0.38, p<0.0001). (b) Age and LA volume indexed to BSA. There was a significant relationship between LA volume and age in G+/LVH+ relatives (blue line, r=0.49, p=0.0003), while there was no significant correlation among HCM probands (red line, r=0.17, p=0.19), G+/LVH- relatives (green line, r=−0.07, p=0.61) and unaffected relatives (black line, r=−0.02, p=0.81).

**Figure 3:**
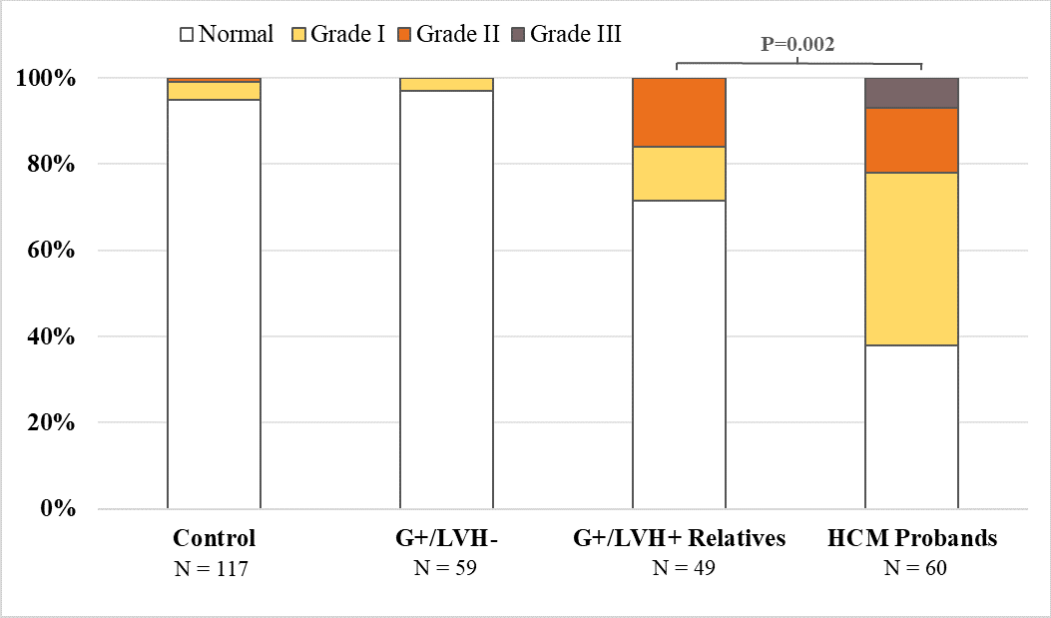
Stacked column bars demonstrating LV diastolic function for each group. HCM probands had significantly worse diastolic function than their G+/LVH+ relatives. Diastolic function was normal in 38% of HCM probands, 71% of G+/LVH+ relatives, 97% of G+/LVH− relatives and 95% of unaffected relatives. P values were adjusted for age, sex and within-family correlation.

Echocardiographic findings are summarised in table 2. There was considerable variation in LVWT (figure 2A) among HCM probands and G+/LVH+ relatives across all ages, but the HCM probands had ~40% greater maximal LVWT overall. HCM probands also exhibited greater variation in LA size and larger mean LA volumes than their G+/LVH+ relatives (figure 2B). Furthermore, HCM probands had higher LV outflow tract gradients, LVWT to LVEDD ratios and more prominent diastolic abnormalities (figure 3) than G+/LVH+ relatives. Cardiac morphology showed a reverse curvature septum in 90% of the HCM probands, compared with 69% of the G+/LVH+ relatives (p=0.019), while 7% of the probands and 22% of the G+/LVH+ relatives had neutral septum morphology (p=0.040). Sigmoid septum morphology or apical hypertrophy was rare in both groups.

**Table 2:** Echocardiographic findings and biomarker analysis

	HCM probands	Relatives			P value HCM probands versus G+/LVH+ relatives	P value G+/LVH− versus unaffected
		G+/LVH+	G+/LVH−	Unaffected		
	(n=60)	(n=49)	(n=59)	(n=117)		
Maximal LVWT, mm	24.9 (0.7)	18.2 (0.8)	9.9 (0.2)	9.5 (0.1)	<0.0001	0.032
LVEDD, mm	42.8 (1.0)	44.0 (1.0)	45.1 (0.7)	46.4 (0.5)	0.409	0.116
LVEDD indexed to BSA, mm/m^2^	21.7 (0.5)	22.4 (0.5)	25.0 (0.6)	25.4 (0.4)	0.249	0.695
LVESD, mm	27.1 (0.8)	27.6 (0.9)	29.0 (0.6)	30.2 (0.4)	0.717	0.059
LVESD indexed to BSA, mm/m^2^	13.7 (0.4)	14.0 (0.4)	16.1 (0.4)	16.42 (0.3)	0.619	0.331
Left atrial diameter, mm	42 (0.9)	40.0 (1.0)	33.6 (0.6)	34.2 (0.4)	0.109	0.406
Left atrial volume indexed to BSA, mm/m^2^	46.0 (2.3)	37.3 (2.4)	24.6 (0.9)	25.2 (0.6)	0.008	0.596
Ratio of LVWT to LVEDD	0.61 (0.02)	0.42 (0.02)	0.23 (0.005)	0.20 (0.004)	<0.0001	0.008
Relative wall thickness	0.51 (0.02)	0.42 (0.02)	0.37 (0.01)	0.34 (0.007)	0.001	0.025
LVEF, %	64.0 (1.5)	66.4 (1.5)	64.9 (1.0)	63.9 (0.7)	0.267	0.350
Peak LV outflow tract gradient, mm Hg	18.3 (3.0)	8.0 (3.1)	7.1 (0.3)	6.7 (0.2)	0.011	0.255
Peak LV outflow tract gradient>30 mm Hg	9 (15%)	0	0	0	0.003	
Tissue Doppler velocities:						
Septal E', cm/s	5.7 (0.3)	8.1 (0.3)	10.1 (0.3)	9.8 (0.2)	<0.0001	0.384
Lateral E', cm/s	8.2 (0.4)	10.7 (0.4)	13.6 (0.4)	13.2 (0.3)	<0.0001	0.395
Septal S', cm/s	7.0 (0.2)	7.7 (0.23)	8.3 (0.20)	8.2 (0.1)	0.025	0.956
Lateral S', cm/s	7.4 (0.3)	9.0 (0.30)	10.2 (0.3)	9.7 (0.2)	0.0001	0.278
Septal E/E' ratio, cm/s	15.6 (0.8)	10.7 (0.86)	8.5 (0.3)	8.7 (0.2)	<0.0001	0.549
Lateral E/E' ratio, cm/s	11.4 (0.7)	8.3 (0.8)	6.4 (0.3)	6.6 (0.2)	0.003	0.761
Septal morphology:						
Reverse curvature septum	54 (90%)	34 (69%)			0.019	
Sigmoid septum	2 (3.3%)	1 (2.0%)			0.488	
Apical hypertrophy	0	3 (6.1%)			0.052	
Neutral septum	4 (6.7%)	11 (22%)			0.040	
Biomarkers						
NT-proBNP, pg/ml	349 (1.2)	116 (1.2)	50.8 (1.1)	34.6 (1.1)	<0.0001	<0.0001
High-sensitivity troponin I, ng/mL	12.3 (1.2)	3.9 (1.2)	1.4 (1.1)	1.1 (1.1)	<0.0001	0.332
PICP, µg/L	103 (4.7)	100 (5.0)	122 (5.5)	118 (3.8)	0.749	0.329

Values are adjusted means (SE) or numbers and proportions (%). P values were adjusted for age, sex and within-family correlation.

BSA, body surface area; LV, left ventricular; LVEDD, left ventricular end-diastolic dimension; LVEF, left ventricular ejection fraction; LVESD, left ventricular end-systolic dimension; LVWT, left ventricular wall thickness; NT-proBNP, N-terminal pro-B natriuretic peptide; PICP, carboxy-terminal propeptide of procollagen type I.

The mean LVWT was slightly higher in G+/LVH− compared with unaffected relatives (9.9±0.2 vs 9.5±0.1, p=0.032), as was the relative wall thickness and ratio of LVWT to LVEDD (table 2). However, when compared across the age span of all study participants, neither LVWT nor LA volumes were substantially different between G+/LVH− and unaffected relatives (figure 1A and B). Similarly, diastolic function (figure 2) did not distinguish G+/LVH− relatives from unaffected relatives.

### Serum biomarkers

NT-proBNP (reflecting haemodynamic stress) and hsTnI (reflecting myocardial injury) levels were significantly higher in HCM probands than in G+/LVH+ relatives (table 2). NT-proBNP levels were higher in G+/LVH− relatives than in unaffected relatives, but hsTnI and PICP (reflecting collagen synthesis) levels were comparable.

### ECG

**Figure 4:**
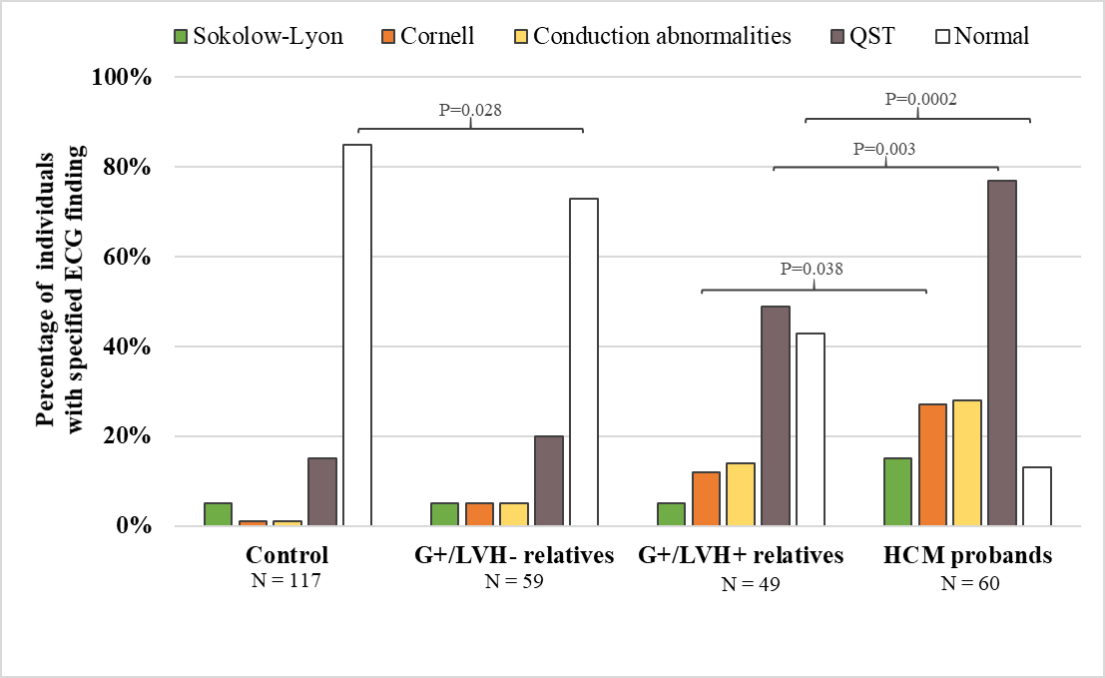
Frequency of ECG abnormalities among each group. ECG abnormalities, including Cornell voltage criteria, Q waves and/or repolarisation abnormalities (QST), were significantly more prevalent among HCM probands than among their G+/LVH+relatives. The G+/LVH- relatives were less likely to have a normal ECG than their unaffected relatives. P-values were adjusted for age, sex and within-family correlation. P-values<0.05 are shown for comparison between HCM probands and G+/LVH+relatives, as well as G+/LVH- relatives vs controls.

ECGs were abnormal in 87% of the HCM probands and 57% of the G+/LVH+ relatives (p=0.0002; figure 4). In contrast, 27% of the G+/LVH− relatives and 15% of the unaffected relatives had abnormal ECGs (p=0.028). Repolarisation abnormalities and/or Q waves (QST) were the most frequently observed abnormalities and were present in 77% of G+/LVH+ probands and 49% of G+/LVH+ relatives (p=0.003). QST was present in 20% of G+/LVH− relatives and 15% of unaffected relatives (p=0.07). The Sokolow–Lyon and Cornell voltage criteria had poor sensitivity for identifying LVH in HCM probands (15% and 27%, respectively) and G+/LVH+ relatives (8.2% and 12%, respectively).

### Penetrance

**Figure 5:**
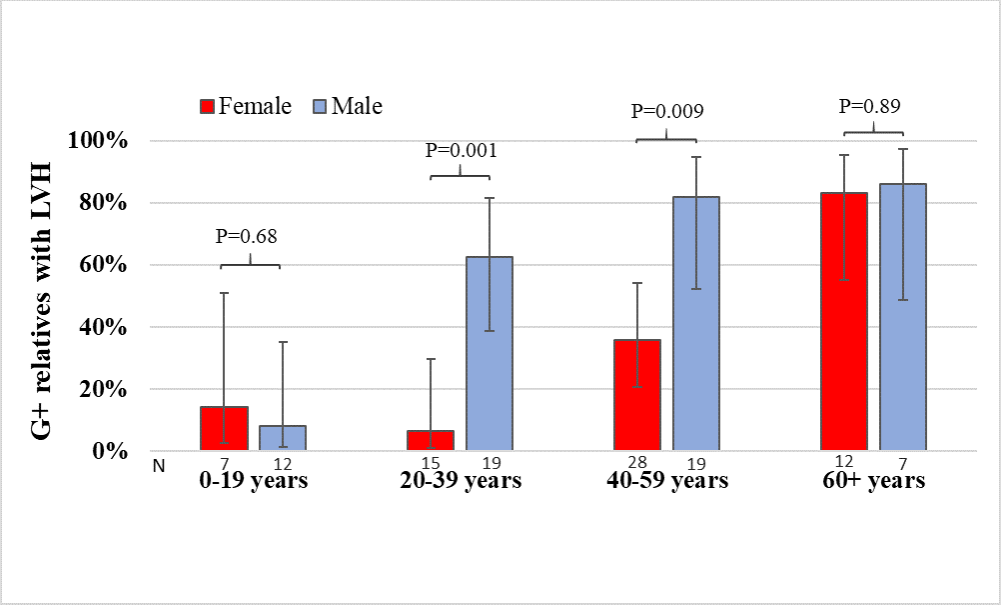
Clustered bar chart demonstrating the proportion (and 95% CI) of *MYBPC3* c.927-2A>G mutation carriers identified through family screening with clinical evidence of LVH, by age and gender.

The penetrance of LVH in G+family members (n=108) was age- and sex-dependent (figure 5). LVH was identified in G+/LVH+ relatives at ages spanning from 11 to 88 years, with a maximal LVWT ranging from 11 to 29 mm. Among G+ relatives aged under 40 years, 39% of men but only 9% of women had LVH (p=0.015). In contrast, after 60 years of age, the prevalence of LVH was high (83%–86%) in both sexes, but not complete. The oldest G+/LVH− male was 70 years old, and the oldest G+/LVH− female was 81 years old.

## Discussion

Analyses of cardiac imaging, ECG and serum biomarker studies in a nationwide cohort provided insights into the clinical expression and penetrance of a highly prevalent Icelandic HCM founder mutation, *MYBPC3* c.927-2A>G. Among the G+ relatives, 45% had LVH at the time of the study. Female mutation carriers developed LVH later in life than males. Before the age of 40 years, the penetrance of LVH was 9% in G+ females in contrast to 39% in G+ males. However, LVH was present in >80% in both sexes after the age of 60 years, indicating a high lifetime penetrance of HCM. Although HCM probands and G+/LVH+ relatives were of comparable ages at the time of the study, HCM probands had a greater burden of disease, including more severe hypertrophy and diastolic dysfunction, more arrhythmias and clinical symptoms, and higher serum troponin and natriuretic peptide levels. G+/LVH− individuals had subtle differences in LV geometry compared with their unaffected relatives, a higher prevalence of ECG abnormalities and a modest, intra-normal elevation of NT-proBNP levels. Collectively, these findings provide evidence for early LV remodelling and haemodynamic stress prior to the development of LVH.

### Age- and sex-related penetrance

There was substantial variation in age of diagnosis and the degree of LVH among the G+ relatives identified through family screening that appears to be associated with both age and sex. LVH was rarely seen in children or adolescents carrying this *MYBPC3* founder mutation. Our results confirm the male predominance and earlier onset of clinical disease previously described in HCM.^14–16^ However, we make a novel observation that this imbalance attenuates with advancing age. Notably, the prevalence of LVH in female mutation carriers increased considerably after the age of 60 years, indicating an approximate 20-year delay in the penetrance of clinically detectable disease for female G+ relatives. Older male and female mutation carriers had an equal prevalence of LVH, indicating that the Icelandic founder mutation is associated with a high lifetime penetrance of HCM. Whether these differences are related to long-term effects of endogenous oestrogens or other factors is unclear.^17^ Additionally, identifying factors that render certain mutation-carriers resilient to developing HCM, even at advanced ages, is a high priority.

### Relatives with LVH

The Icelandic G+/LVH+ relatives diagnosed through family screening had less advanced disease (few symptoms, lower maximal LVWT, smaller LA, less obstructive physiology and less prevalent diastolic dysfunction) despite older age at diagnosis compared with HCM probands. In addition, the prototypical HCM morphology of reverse curvature septal hypertrophy was less common in G+/LVH+ relatives than in the probands (p=0.019). Assessments of ECG abnormalities, systolic and early diastolic tissue Doppler velocities, and hsTnI and NT-proBNP confirmed milder clinical expression in G+/LVH+ relatives than in HCM probands.

These findings are a vivid demonstration of the phenotypic variability possible with a single pathogenic mutation. Factors that drive the marked heterogeneity in disease expression and clinical outcomes are poorly understood. By anchoring to a single pathogenic variant, further detailed studies of founder populations will provide valuable opportunities to better refine genotype–phenotype correlations and to better understand features that contribute to phenotypic heterogeneity.

### Comparisons with other studies of *MYBPC3* founder mutations

Multiple founder mutations have been identified in *MYBPC3,*^1 4–7^ that all cause premature truncation and haploinsufficiency of myosin-binding protein in sarcomeres. The effects of three Dutch founder mutations (46% *MYBPC3* c.2373dupG; 32% *MYBPC3* c.2827C>T; 22% *MYBPC3* c.2864_2865delCT) were characterised in 134 HCM probands and 54 G+/LVH+ relatives, identified by family screening,^2^ similar to our approach. An Italian study of the *MYBPC3* p.F305Pfs*27 founder mutation^1^ characterised 19 HCM probands and 29 G+/LVH+ relatives who were identified either by family screening (72%) or clinical presentation (28%). Across these founder mutation populations, HCM probands had a similar predominance of male sex, age of clinical diagnosis, maximum LVWT and prevalence of NYHA ≥2 functional class. Among Dutch and Icelandic G+/LVH+ relatives diagnosed by family screening, clinical features were also remarkably consistent and milder than those in HCM probands (table 3). Although previous studies underscore the heterogeneous nature of HCM even among individuals with identical mutations^18^ and attribute these differences to genetic background and/or modifying environmental factors and lifestyles, we observed remarkable phenotypic similarities among cohorts of HCM probands and G+/LVH+ relatives with different *MYPBC3* founder mutations. Given the distinct genetic backgrounds of the Dutch and Icelandic populations, differences in environments and lifestyle, these data provide evidence for relatively consistent direct or proximal consequences of *MYBPC3* haploinsufficiency. However, the concordance between G+/LVH+ relatives may also indicate the presence of other common genetic modifiers that attenuates disease expression (relative to probands) despite the presence of pathogenic genotypes. Given the shared molecular mechanisms between different truncating *MYBPC*3 founder mutations and the statistical power achieved by combining multiple families with these mutations, collaborative genomic analyses may uncover important deleterious or protective alleles.

**Table 3:** Comparison of *MYBPC3* founder mutations in three populations

	Icelandic probands (n=60)	Dutch probands (n=134)*	Italian probands (n=19)†	Icelandic G+/LVH+ relatives (n=49)	Dutch G+/LVH+ relatives (n=54)*	Italian G+/LVH+ relatives (n=29)†
Mutation	c.927-2A>G	c.2373dupG (46%), c.2827C>T (32%), c.2864_2865 delCT (22%)	p.F305Pfs*27	c.927-2A>G	c.2373dupG (46%), c.2827C>T (32%), c.2864_2865 delCT (22%)	p.F305Pfs*27
Diagnosed by family screening				100%	100%	72%
Male (%)	67%	67%	74%	55%	57%	59%
Age at diagnosis	41±14	44±14	36±16	50±19	47±16	44±19
LVWT, mean (mm)	25	20	23	18	16	19
LVOT gradient>30 mm Hg (%)	15%	28%	16%	0%	4%	14%
Left atrial diameter, mean (mm)	42	45	49	40	40	45
Diastolic dysfunction (%)	62%	56%	N/A	29%	38%	N/A
NYHA class≥2 (%)	48%	48%	58%	0%	8%	28%
Atrial fibrillation (%)	18%	21%	21%	8%	7%	14%
SCD/aborted SCD (%)	8%	14%	32%	0%	4%	7%
ICD (%)	18%	23%	58%	0%	13%	14%

*Clinical data from Dutch subjects with *MYBPC3* founder mutations reported in.^2^

†Clinical data from Italian subjects with a *MYBPC3* founder mutation reported in.^1^

G+, genotype-positive; HCM, hypertrophic cardiomyopathy; ICD, implantable cardioverter defibrillator; LVH, left ventricular hypertrophy; LVOT, left ventricular outflow tract; LVWT, left ventricular wall thickness; MYBPC3, myosin-binding protein C; NYHA, New York Heart Association; SCD, suden cardiac death.

### Preclinical HCM and early phenotypes

Early morphological and electrophysiological phenotypes reported among sarcomere mutation carriers with normal LVWT include subtle changes in LV cavity size and geometry, impaired LV relaxation^19–22^ and ECG abnormalities.^11^ As most of these studies analysed small and/or heterogeneous cohorts, interpretation of these findings has been difficult.^20 23^ Our analyses of preclinical HCM subjects with a shared founder *MYBPC3* mutation advances these findings. In all, 59 G+ relatives had no diagnostic criteria for HCM, including 10 individuals over 50 years of age. LVWT and LA dimensions were normal, although the ratio of LVWT to LVEDD and the relative wall thickness was slightly higher in G+/LVH− relatives compared with unaffected relatives. Diastolic function parameters were normal in 97% of G+/LVH and 95% of unaffected relatives, and no significant difference was detected in any parameter of LV relaxation and estimation of LV filling pressures. However, while NT-proBNP levels did not discriminate preclinical (G+/LVH−) subjects from controls in prior studies,^12 13 20 23 24^ levels were higher in our G+/LVH− compared with unaffected relatives, although still within the normal range. ECG abnormalities, predominantly Q waves and repolarisation changes, were more prevalent among G+/LVH− individuals than among unaffected relatives (27% vs 15%; p=0.028) and may reflect early manifestations of HCM,^11^ although with low positive predictive value. Overall, the composite phenotypic manifestations of these 59 G+/LVH− adults were subtle. We suggest that analyses of the impact of genetics and lifestyle, and determination of how early phenotypic changes relate to development of clinically overt HCM are worthy of further study.

### Limitations

This was a cross-sectional study, and participants were only screened for the *MYBPC3* c.927-2A>G mutation. Therefore, we cannot exclude the possibility of additional variants that may contribute to clinical variability. This study involved families with at least one member with overt HCM due to the *MYBPC3* c.927-2A>G founder mutation, while we estimate that~85% of the founder mutation carriers in Iceland or approximately 950 individuals remain clinically undetected with unknown clinical status.^3^ Similar to most HCM studies, this study is biassed towards individuals with clinical HCM diagnosis and their first-degree relatives, and it might be difficult to extrapolate the results SE findings to the whole founder population of Iceland. A larger population-based study would add to the current knowledge of disease penetrance and disease expression and possibly identify either genetic or environmental modifiers that have significant impact on disease manifestation.

## Conclusions

Through comprehensive family screening of a nationwide cohort of Icelandic individuals carrying the same *MYBPC3* founder mutation, we demonstrated that HCM occurs at an earlier age in men, but the lifetime penetrance appears to be high and equivalent in both men and women. Relatives diagnosed with HCM in the context of family screening were older at initial evaluation and had a milder clinical phenotype than HCM probands. Furthermore, phenotypic manifestations of the Icelandic *MYBPC3* founder mutation were remarkably similar to other distinct founder populations with truncating *MYBPC3* variants suggesting a common effect in the context of haploinsufficiency in the myosin-binding protein. Finally, we found a higher prevalence of abnormal ECGs and subtle differences in LV geometry in G+/LVH– subjects compared with unaffected relatives. Collaborative longitudinal clinical and genetic studies are needed to identify factors that modify disease expression, drive phenotypic progression and lead to adverse clinical outcomes.

## References

[1] Calore C, De Bortoli M, Romualdi C, et al A founder MYBPC3 mutation results in HCM with a high risk of sudden death after the fourth decade of life. J Med Genet 2015;52:338–47. 10.1136/jmedgenet-2014-102923 25740977

[2] van Velzen HG, Schinkel AFL, Oldenburg RA, et al Clinical Characteristics and Long-Term Outcome of Hypertrophic Cardiomyopathy in Individuals With a MYBPC3 (Myosin-Binding Protein C) Founder Mutation. Circ Cardiovasc Genet 2017;10 10.1161/CIRCGENETICS.116.001660 28794111

[3] Adalsteinsdottir B, Teekakirikul P, Maron BJ, et al Nationwide study on hypertrophic cardiomyopathy in Iceland: evidence of a MYBPC3 founder mutation. Circulation 2014;130:1158–67. 10.1161/CIRCULATIONAHA.114.011207 25078086

[4] Jääskeläinen P, Heliö T, Aalto-Setälä K, et al Two founder mutations in the alpha-tropomyosin and the cardiac myosin-binding protein C genes are common causes of hypertrophic cardiomyopathy in the Finnish population. Ann Med 2013;45:85–90. 10.3109/07853890.2012.671534 22462493

[5] Christiaans I, Nannenberg EA, Dooijes D, et al Founder mutations in hypertrophic cardiomyopathy patients in the Netherlands. Neth Heart J 2010;18:248–54. 10.1007/BF03091771 20505798 PMC2871745

[6] Oliva-Sandoval MJ, Ruiz-Espejo F, Monserrat L, et al Insights into genotype-phenotype correlation in hypertrophic cardiomyopathy. Findings from 18 Spanish families with a single mutation in MYBPC3. Heart 2010;96:1980–4. 10.1136/hrt.2010.200402 21088121

[7] Girolami F, Olivotto I, Passerini I, et al A molecular screening strategy based on beta-myosin heavy chain, cardiac myosin binding protein C and troponin T genes in Italian patients with hypertrophic cardiomyopathy. J Cardiovasc Med 2006;7:601–7. 10.2459/01.JCM.0000237908.26377.d6 16858239

[8] Lang RM, Badano LP, Mor-Avi V, et al Recommendations for cardiac chamber quantification by echocardiography in adults: an update from the American Society of echocardiography and the European association of cardiovascular imaging. J Am Soc Echocardiogr 2015;28:1–39. 10.1016/j.echo.2014.10.003 25559473

[9] Nagueh SF, Smiseth OA, Appleton CP, et al Recommendations for the evaluation of left ventricular diastolic function by echocardiography: an update from the American Society of echocardiography and the European association of cardiovascular imaging. Eur Heart J Cardiovasc Imaging 2016;17:1321–60. 10.1093/ehjci/jew082 27422899

[10] Binder J, Ommen SR, Gersh BJ, et al Echocardiography-guided genetic testing in hypertrophic cardiomyopathy: septal morphological features predict the presence of myofilament mutations. Mayo Clin Proc 2006;81:459–67. 10.4065/81.4.459 16610565

[11] Lakdawala NK, Thune JJ, Maron BJ, et al Electrocardiographic features of sarcomere mutation carriers with and without clinically overt hypertrophic cardiomyopathy. Am J Cardiol 2011;108:1606–13. 10.1016/j.amjcard.2011.07.019 21943931 PMC3215918

[12] Ho JE, Shi L, Day SM, et al Biomarkers of cardiovascular stress and fibrosis in preclinical hypertrophic cardiomyopathy. Open Heart 2017;4:e000615 10.1136/openhrt-2017-000615 29177058 PMC5687543

[13] Ho CY, López B, Coelho-Filho OR, et al Myocardial fibrosis as an early manifestation of hypertrophic cardiomyopathy. N Engl J Med 2010;363:552–63. 10.1056/NEJMoa1002659 20818890 PMC3049917

[14] Olivotto I, Maron MS, Adabag AS, et al Gender-Related differences in the clinical presentation and outcome of hypertrophic cardiomyopathy. J Am Coll Cardiol 2005;46:480–7. 10.1016/j.jacc.2005.04.043 16053962

[15] van Velzen HG, Schinkel AFL, Baart SJ, et al Outcomes of contemporary family screening in hypertrophic cardiomyopathy. Circ Genom Precis Med 2018;11:e001896 10.1161/CIRCGEN.117.001896 29661763

[16] Terauchi Y, Kubo T, Baba Y, et al Gender differences in the clinical features of hypertrophic cardiomyopathy caused by cardiac myosin-binding protein C gene mutations. J Cardiol 2015;65:423–8. 10.1016/j.jjcc.2014.07.010 25123604

[17] Haines CD, Harvey PA, Luczak ED, et al Estrogenic compounds are not always cardioprotective and can be lethal in males with genetic heart disease. Endocrinology 2012;153:4470–9. 10.1210/en.2012-1391 22778230 PMC3423614

[18] Page SP, Kounas S, Syrris P, et al Cardiac myosin binding protein-C mutations in families with hypertrophic cardiomyopathy: disease expression in relation to age, gender, and long term outcome. Circ Cardiovasc Genet 2012;5:156–66. 10.1161/CIRCGENETICS.111.960831 22267749

[19] Michels M, Soliman OII, Kofflard MJ, et al Diastolic abnormalities as the first feature of hypertrophic cardiomyopathy in Dutch myosin-binding protein C founder mutations. JACC Cardiovasc Imaging 2009;2:58–64. 10.1016/j.jcmg.2008.08.003 19356534

[20] Ho CY, Day SM, Colan SD, et al The burden of early phenotypes and the influence of wall thickness in hypertrophic cardiomyopathy mutation carriers: findings from the HCMNet study. JAMA Cardiol 2017;2:419–28. 10.1001/jamacardio.2016.5670 28241245 PMC5541992

[21] Ho CY, Sweitzer NK, McDonough B, et al Assessment of diastolic function with Doppler tissue imaging to predict genotype in preclinical hypertrophic cardiomyopathy. Circulation 2002;105:2992–7. 10.1161/01.cir.0000019070.70491.6d 12081993

[22] Gandjbakhch E, Gackowski A, Tezenas du Montcel S, et al Early identification of mutation carriers in familial hypertrophic cardiomyopathy by combined echocardiography and tissue Doppler imaging. Eur Heart J 2010;31:1599–607. 10.1093/eurheartj/ehq101 20439259

[23] Silva D, Madeira H, Almeida A, et al Tissue Doppler imaging and plasma N-terminal probrain natriuretic peptide for the identification of hypertrophic cardiomyopathy mutation carriers. Am J Cardiol 2013;112:996–1004. 10.1016/j.amjcard.2013.05.039 23831167

[24] Ho CY, Abbasi SA, Neilan TG, et al T1 measurements identify extracellular volume expansion in hypertrophic cardiomyopathy sarcomere mutation carriers with and without left ventricular hypertrophy. Circ Cardiovasc Imaging 2013;6:415–22. 10.1161/CIRCIMAGING.112.000333 23549607 PMC3769196

